# Intraoperative infusion of branched-chain amino acids in patients undergoing gastrointestinal tumor surgery

**DOI:** 10.1186/s12957-015-0751-y

**Published:** 2015-12-15

**Authors:** Qiwei Wu, Yanying Zhang, Yan Yang, Shengjin Ge, Zhanggang Xue

**Affiliations:** Department of Anaesthesia, Zhongshan Hospital, Fudan University, No. 180 Fenglin Road, Shanghai, 200032 China; Department of Anaesthesiology, Shanghai Medical College, Fudan University, Shanghai, 200032 China

**Keywords:** Metabolism-glucose, Metabolism-lipid, Temperature-metabolism

## Abstract

**Background:**

The purpose of this study is to investigate the effects of intraoperative infusion of branched-chain amino acids (BCAA) in patients undergoing gastrointestinal tumor surgery.

**Methods:**

Sixty-one patients with gastrointestinal tumors undergoing gastrointestinal surgery were enrolled and randomly assigned to receive an intraoperative infusion of 3-compound BCAA solution (*N* = 20), amino acids (AA) solution (*N* = 21), or normal saline (NS) (*N* = 20). Nasopharyngeal temperature, blood glucose (BG), plasma insulin, and blood free fatty acids (FFA) concentrations were measured at 30 min before and 10 min after induction (*T*_0_,*T*_1_), 30 min and 2 h after skin incision (*T*_2_,*T*_3_), and 1 h after tracheal extubation (*T*_4_). Intensity of shivering and pain was accessed at 1 h after extubation.

**Results:**

The temperature in the BCAA and AA group was significantly higher than that in the NS group at *T*_4_ (*P* = 0.014 and 0.033). The incidence of shivering in the BCAA and AA group was significantly lower than in the NS group (*P* = 0.027 and 0.012). BG increased in AA group at *T*_3_ and *T*_4_ (*P* = 0.001 and 0.045). The plasma insulin concentration increased in the BCAA and AA group from *T*_1_ to *T*_3_. The plasma FFA concentrations in the BCAA group were lower than in the AA and NS group from *T*_2_ to *T*_4_.

**Conclusions:**

Intraoperative BCAA and AA infusion alleviated postoperative hypothermia and shivering. BCAA infusion also inhibited fat mobilization, without adversely affecting blood glucose.

**Trial registration:**

ChiCTR-TRC-14004668

**Electronic supplementary material:**

The online version of this article (doi:10.1186/s12957-015-0751-y) contains supplementary material, which is available to authorized users.

## Background

During anesthesia and surgery, redistribution hypothermia occurs as peripheral vessels dilate, and impaired hypothalamic thermoregulation further worsens the hypothermia [1, 2]. Thereafter, core body temperature gradually declines as heat is lost from the surface of the body, and the production of heat slows [3–6]. Furthermore, the stress of surgery and anesthesia leads to a series of changes at the metabolic level and in the neuro-endocrine axis [2, 7]. Catabolism exceeds anabolism, thereby increasing the physiological burden of patients undergoing surgery.

Unlike other amino acids (AAs) that are metabolized dominantly in the liver, the branched-chain amino acids (BCAA), isoleucine, leucine, and valine are uniquely metabolized primarily in the skeletal muscle. Moreover, BCAAs can promote anabolism without placing additional burdens on the liver. In previous studies, infusions of compound intravenous AA solutions have been shown to exhibit a thermogenic effect during anesthesia [8–10]. Therefore, we hypothesized that intraoperative infusion of BCAA instead of AAs could provide even more benefit to patients. The objective of our study was to investigate the effects of intraoperative infusion of BCAA on body temperature, carbohydrate metabolism, and lipid metabolism in patients undergoing gastrointestinal (GI) tumor surgery.

## Methods

### Patients

The study was approved by the Ethics Committee of Zhongshan Hospital, Fudan University (B2014-013) and registered at www.chictr.org.cn (ChiCTR-TRC-14004668). Written informed consent was obtained from all patients. We screened a total of 69 consecutive patients (aged 18–65 years and American Society of Anesthesiologist physical status classes I–II) scheduled to undergo GI surgery (stomach or colorectal) at Zhongshan Hospital from May to July 2014. Patients were excluded if they had a serum albumin <35 g·L^−1^, anemia (hemoglobin <100 g·L^−1^), or evidence of a major medical condition (such as diabetes mellitus, thyroid disease, hepatic disease, or renal disease). They were also excluded if they were receiving drugs known to have metabolic effects, such as corticosteroids or β-blockers. Only 61 patients fulfilled all criteria. They were randomly assigned at a 1:1:1 ratio to one of three groups using random number method: BCAA infusion group (compound amino acid injection, BBCA Pharmaceutical Co. Ltd., Anhui, China; see Appendix), AA infusion group (Aminoplasmal, B. Braun Melsungen AG, Jiangsu, China; see in Appendix), and normal saline (NS) infusion group (normal saline, Baxter Healthcare Co. Ltd., Shanghai, China). All patients underwent standard GI preoperative procedures, including overnight fasting. Patients scheduled for colorectal operations also underwent bowel-cleansing procedures the day before surgery. No premedications were given except intestinal antibacterial drugs, which were started 3 days preoperatively.

### Anesthesia

Before the induction of general anesthesia, each patient received an epidural catheter between *T*_8_ and *T*_12_, a central venous catheter, and a radial artery catheter. Hemodynamic monitoring was performed using a three-lead electrocardiogram monitor, finger pulse oximetry, and continuous arterial pressure measurement via the radial artery catheter.

General anesthesia was induced in all patients with fentanyl 3 μg·kg^−1^, propofol 2 mg·kg^−1^, and lidocaine 60 mg, and rocuronium 0.6 mg·kg^−1^ was administered to facilitate intubation. After tracheal intubation, mechanical ventilation was initiated with 50 % oxygen: 50 % air-gas mixture, and ventilation was adjusted to maintain the end-tidal CO_2_ at 30–40 mmHg. After induction, 6–10 mL of 0.5 % bupivacaine was administered in increments through the epidural catheter, and epidural anesthesia was maintained with intermittent boluses of 3–5 mL every hour during the operation. General anesthesia was maintained with sevoflurane at a minimum alveolar concentration of 0.8–0.9. Boluses of phenylephrine or ephedrine were administered if necessary to maintain the heart rate and arterial pressure within ±30 % of baseline. Each patient received 0.125 % bupivacaine and fentanyl 2 μg·mL^−1^ for patient-controlled epidural analgesia postoperatively.

### Experimental protocol

The operating room temperature was maintained at 21–23 °C. No other perioperative warming strategies were used except the blood products that were warmed to 42 °C if a transfusion was needed. A forced-air warming system would be used as a rescue method, if the body core temperature dropped below 35 °C. All patients were covered with two layers of quilt postoperatively.

The AA infusion was diluted to 4.26 % with normal saline. The total dose of BCAA was calculated as 5.6 mL·kg^−1^·h^−1^ according to the basal metabolic rate, 4 kJ·kg^−1^·h^−1^. The intervention fluid (BCAA, AA, or NS) was infused at a rate of 5.6 mL·kg^−1^·h^−1^. In addition, Lactated Ringer’s solution (lot no. S1401082; Baxter Healthcare Co. Ltd., Shanghai, China) was administered at 4 mL·kg^−1^·h^−1^ as the basal fluid infusion. All intervention fluids were infused via a central venous catheter, using a Graseby 3500 pump (SIMS Graseby Ltd., Watford, UK). The infusions were initiated at the beginning of induction and discontinued when the patient was extubated.

The BCAA, AA, and NS solutions were prepared by a specified investigator. The other investigators, all health-care providers, and all patients were blinded to the preparation procedure and were thereby unaware of the group assignments.

### Measurements

The core temperature was measured continuously using a nasopharyngeal temperature probe (Datex-Ohmeda Division, Instrumentarium Corp., Helsinki, Finland) which was inserted from the nostril with the distance from the philtrum to the tragus. Blood samples were collected 30 min before induction (*T*_0_), 10 min after induction (*T*_1_), 30 min and 2 h after skin incision (*T*_2_ and *T*_3_), and 1 h after extubation (*T*_4_). Each blood sample was transferred immediately to an EDTA tube and centrifuged at 4 °C until analyzed within 24 h by an automatic biochemical analyzer (7080, Hitachi, Japan) and automatic electrochemiluminescence analyzer (Cobas e602, Roche, Swiss). At the same time points, the blood glucose concentration was measured by a glucose analyzer (One Touch Sure-step; LifeScan, Johnson & Johnson Medical Co. Ltd., Shanghai, China) using a sample of blood obtained from the fingertips. At 1 h after tracheal extubation, the intensity of shivering was assessed by a single investigator (who was the same for each patient) using the Wrench grade for post-anesthetic shivering [11] (Additional file 1: Table S1), and the severity of pain was assessed using a visual analog scale (with 0 =no pain and 10 =worst pain imaginable) [12].

### Sample size estimation and statistical analysis

In this study, the primary outcome measure was nasopharyngeal temperature, and the secondary outcome measures were blood glucose concentration and circulating concentrations of plasma insulin and free fatty acids (FFA). PASS 11.0 (NCSS, Kaysville, Utah, USA) were used for sample size estimation and power calculation. In previous studies [10], the standard deviation of body temperature was approximately 0.1–0.2 °C, and the expected difference between groups was approximately 0.3–0.5 °C; therefore, less than nine patients in each group were calculated to be sufficient for this outcome (power 80 %, *α* = 0.05). On the basis of an expected difference in blood glucose concentration of 1 mmol·L^−1^ between the two groups (standard deviation =1 mmol·L^−1^; power 80 %, *α* = 0.05), 17 patients in each group were calculated to be sufficient for this study [10, 13, 14]. Thus, 20 patients in each group were deemed sufficient for this study. Statistical analyses were performed using SPSS 20.0 (SPSS, Inc.) software. Categorical variables, including gender and type of operation, were analyzed using *χ*^2^ or Fisher’s exact test. Continuous variables were analyzed using two-tailed general linear model repeated measures analysis of variance. Probability (*p*) values <0.05 were considered statistically significant.

## Results

There were no baseline differences among the three groups for gender, age, body mass index (BMI), type of operation, duration of surgery, and anesthesia (Table 1). The severity of pain 1 h after surgery between the two groups did not reach statistical significance. No patient received a blood transfusion in this study. The mean postoperative hospitalization days were 7.9 ± 1.8, 8.1 ± 1.9, and 8.6 ± 2.4 days for the BCAA, AA, and NS groups, respectively. Only one patient in the AA group suffered from bowel leak in hospitalization within 30 days after operation. The continuous data were normally distributed, with homogeneity of variance (data not shown). The baseline data (30 min before anesthesia) for core temperature, blood glucose, plasma insulin, and FFA level were not significantly different among the three groups (Table 2).

**Table 1:** Baseline characteristics of the three groups

	BCAA (*N* = 20)	AA (*N* = 21)	NS (*N* = 20)	*P*
Gender				0.509
Male	13 (65.0)	10 (47.6)	12 (60.0)	
Female	7 (35.0)	11 (52.4)	8 (40.0)	
Age (years)	56.5 (18 to 65)	58 (40 to 68)	52 (34 to 65)	0.313
BMI (kg/m^2^)	23.62 ± 2.33	23.32 ± 3.02	22.83 ± 2.33	0.594
Surgery type^a^				0.710
Gastric	8 (40.0)	10 (47.6)	7 (35.0)	
Colorectal	12 (60.0)	11 (52.4)	13 (65.0)	
Duration of surgery (min)	146.75 ± 2.92	147.14 ± 22.89	137.37 ± 36.41	0.428
Total volume of fluid (mL)	2615.00 ± 241.21	2504.76 ± 423.64	2457.90 ± 287.36	0.006
Amino acids				
Non-BCAA (g)	0	26.35 ± 5.55	0	
BCAA (g)	38.62 ± 7.75	11.29 ± 2.38	0	

Data are mean ± standard deviation or number (percentage)

*Abbreviations*: *BCAA* branched-chain amino acids, *BMI* body mass index, *NS* normal saline, AA amino acid

^a^Surgeries were conducted open

**Table 2:** Changes in nasopharyngeal temperature and plasma concentrations of glucose, insulin, and free fatty acids

Factors	Variables	BCAA (*N* = 20)	AA (*N* = 21)	NS (*N* = 20)	P*
*T* (°C)	*T*_0_	36.77 ± 0.37	36.56 ± 0.35	36.61 ± 0.37	0.177
	*T*_1_	36.51 ± 0.31	36.40 ± 0.35	36.35 ± 0.39	0.335
	*T*_2_	36.21 ± 0.35	36.26 ± 0.35	36.16 ± 0.47	0.709
	*T*_3_	35.89 ± 0.42	36.17 ± 0.36	35.82 ± 0.56	*0.040*
	*T*_4_	36.39 ± 0.40	36.34 ± 0.34	36.05 ± 0.49	*0.029*
BG (mmol·L^−1^)	*T*_0_	5.12 ± 0.89	5.11 ± 0.45	4.83 ± 0.92	0.436
	*T*_1_	5.47 ± 0.78	5.48 ± 0.56	5.15 ± 1.02	0.349
	*T*_2_	5.87 ± 0.92	6.32 ± 0.61	5.92 ± 1.39	0.297
	*T*_3_	5.96 ± 0.95	7.61 ± 1.24	6.05 ± 2.07	*0.001*
	*T*_4_	6.09 ± 1.33	7.21 ± 1.28	7.05 ± 1.84	*0.045*
Insulin (μU·mL^−1^)	*T*_0_	4.38 ± 2.40	4.41 ± 3.69	3.34 ± 2.50	0.276
	*T*_1_	6.85 ± 4.46	6.87 ± 5.46	3.17 ± 3.03	*0.016*
	*T*_2_	6.86 ± 4.78	16.12 ± 12.25	2.18 ± 2.05	*0.000*
	*T*_3_	12.72 ± 7.83	28.89 ± 21.10	2.57 ± 1.62	*0.000*
	*T*_4_	10.56 ± 5.53	8.55 ± 6.09	4.03 ± 2.98	*0.001*
FFA (mmol·L^−1^)	*T*_0_	0.86 ± 0.30	0.80 ± 0.37	0.96 ± 0.29	0.288
	*T*_1_	0.66 ± 0.26	0.59 ± 0.25	0.78 ± 0.26	0.075
	*T*_2_	0.47 ± 0.14	0.39 ± 0.16	0.59 ± 0.23	*0.003*
	*T*_3_	0.42 ± 0.19	0.89 ± 0.30	0.74 ± 0.37	*0.000*
	*T*_4_	0.37 ± 0.19	0.58 ± 0.24	0.86 ± 0.40	*0.000*

Data are represented as mean ± standard deviation. Italicized texts indicate significance

*Abbreviations*: *T* nasopharyngeal temperature, *BG* blood glucose, *FFA* free fatty acids, *BCAA* branched-chain amino acids, *NS* normal saline, *AA* amino acids, *T*_*0*_ 30 min before induction, *T*_*1*_ 10 min after induction (before skin incision), *T*_*2*_ and *T*_*3*_ 30 min and 2 h after skin incision, *T*_*4*_ 1 h after extubation

*P mean value among three groups (one way ANOVA)

The patients’ core temperature in both the BCAA and NS groups slowly decreased during surgery while none dropped below 35 °C. However, the mean temperature in the BCAA and AA groups was significantly higher at 1 h after extubation than in the NS group (36.39, 36.34 vs 36.05, *P* = 0.029, power = 80.76 %) (Table 2). There was no significant difference in incidence and intensity of shivering (grade 0 vs grade ≥1) between the BCAA and AA group. BCAA and AA groups were significantly different from NS group (*P* values were 0.027 and 0.012, respectively Table 3).

**Table 3:** Shivering grades at 1 h after extubation

Group	Grade				
	0	1	2	3	4
BCAA (*N* = 20)	14	5	1	0	0
AA (*N* = 21)	15	4	2	0	0
NS (*N* = 20)	7	4	5	3	1

In comparison of shivering grades (grade 0 vs grade ≥1), BCAA and AA groups were significantly different from the NS group (*P* value were 0.027 and 0.012, respectively.). There was no significant difference between BCAA and AA groups (*P* = 0.920)

*Abbreviations*: *BCAA* branched-chain amino acids, *AA* amino acids, *NS* normal saline

Blood glucose concentrations increased after the induction of anesthesia in all three groups. There were no significant differences between the BCAA and NS groups over time, although the values in the BCAA group seemed to be more stable than those in the NS group. BG in the AA group peaked at *T*_3_ (7.61 mM) and were higher at *T*_3_ and *T*_4_ comparing with the BCAA group (*P* = 0.001 and 0.045, power = 99.96). The plasma insulin concentration of BCAA and AA groups began to increase at *T*_1_ and peaked at *T*_3_. The plasma insulin in the NS group stayed in baseline level during surgery. The insulin values were significantly higher in BCAA and AA patients than in NS patients from *T*_1_ to *T*_4_ (all *P* < 0.05, power = 99.80, Table 2). Moreover, in AA patients, the insulin level increased to 28.89 μU/mL at *T*_3_.

The FFA concentrations in the NS and AA groups decreased at the beginning of anesthesia, hit a nadir at 30 min after skin incision, and then slowly increased until 1 h after extubation. By contrast, plasma FFA concentrations in the BCAA group decreased from 10 min after induction to the end of surgery and remained low thereafter (Table 2).

## Discussion

Nutrients such as glucose, fatty acids, and AAs are not usually administered intraoperatively because of the stress response that typically accompanies anesthesia and surgery [15]. However, recent studies have demonstrated that administration of AAs can stimulate energy expenditure and enhance thermogenesis during surgery and anesthesia [8, 9, 16–19]. Furthermore, the optimal composition of intraoperative AAs remains poorly understood. Our current results suggest that BCAA administration can alleviate postoperative shivering. Moreover, BCAA infusion had little influence on the blood glucose concentration, thereby suggesting that it is safe during anesthesia and surgery from this perspective.

In our previous study of patients undergoing surgery under general anesthesia combined with epidural block, the thermogenic effects of intraoperative AAs infusion were not significant in the early part of surgery; they were most obvious at 2 h after the induction of anesthesia [14]. This was consistent with our results. The current study, which was the first randomized controlled trial to investigate the thermogenic effects of BCAA in patients, showed that BCAA infusion also had a delayed thermogenic response. Furthermore, the BCAA infusion was also noted to decrease the incidence of postoperative shivering as AA infusion did.

Previous studies in humans [20], as well as in an animal model [10], have reported substantial increases in blood glucose and insulin concentrations during the administration of AAs. This phenomenon was reproducible in our study. Although our current results demonstrated a statistically significant increase in blood glucose while infusing BCAA, the increase was not as dramatic as that in the AA group. Moreover, in the AA group, insulin level increased significantly as well. Previous studies demonstrated that plasma AA concentration was closely associated with insulin resistance [21–24]. Several experiments on human and rats suggested that the increased plasma AA concentration reduced insulin-induced peripheral glucose disposal by interfering with muscle glucose transport and phosphorylation [25, 26]. This phenomenon was observed in the present study. In the BCAA and AA groups, although plasma insulin level largely increased after BCAA or AA infusion, the plasma glucose concentration was still upregulated. In other words, glucose utilization decreased. In clinical practice, a blood glucose concentration of 6–10 mmol·L^−1^ would be considered acceptable. Our study demonstrated that BCAA infusion had less effect on blood glucose than AA did.

As a consequence of the endocrine response to stress, lipid mobilization is enhanced [27]. In the current study, the FFA concentration in the BCAA group declined during surgery, whereas the FFA concentration in the NS group decreased at *T*_2_ but subsequently rose at *T*_3_. This may reflect a decreased lipid mobilization effect of BCAA. Although detailed mechanisms must still be uncovered, one explanation may be that lipolysis is suppressed by the enhanced insulin secretion trigged by BCAA [28]. In the AA group, FFA concentration was unstable during surgery. This might due to the influence of complicated component of AA on lipid mobilization.

Limitations of this study should be clarified. Although the sample size estimation showed that our number of subjects was adequate for statistical analysis, our sample size was small and validation is required in a larger population. Furthermore, we did not obtain a muscle biopsy to investigate the direct results of BCAA metabolism in the skeletal muscle, and further studies, including invasive approaches for studying BCAA metabolism, are still needed. Also, the long-term complications of patients who underwent GI surgery were not included in this study. It remains unknown whether the effect of BCAA on insulin induction could have detrimental effect on type 2 diabetes or even pre-diabetic patients.

## Conclusions

In conclusion, our findings indicate that an intraoperative infusion of BCAA can alleviate hypothermia and the intensity of shivering after GI surgery. It can also inhibit fat mobilization, without adversely affecting blood glucose concentrations in our cohort. Further multicenter external validation is needed.

## Statement of ethics and consent

The study was approved by the Ethics Committee of Zhongshan Hospital, Fudan University (B2014-013) and registered at www.chictr.org.cn (ChiCTR-TRC-14004668). Written informed consent was obtained from all patients to participate under the “Ethics, consent, and permissions” heading and to publish from the participant to report individual patient data.

## Appendix

**Table 4:** Composition of the 3-compound branched-chain amino acids injection (per 1000 ml)

Amino acids	Values (g)
l-isoleucine	13.50
l-leucine	16.50
l-valine	12.60
Total amino acids	42.60

Osmotic pressure: approximately 382 mOsm/L

**Table 5:** Composition of the compound amino acids injection (Aminoplasmal, per 1000 ml)

Amino acids	Values (g)
l-Alanine	8.30
l-Valine	10.60
l-Leucine	13.60
l-Isoleucine	8.80
l-Phenylalanine	1.60
l-Tryptophane	1.50
l-Methionine	1.20
l-Proline	7.10
Glycine	6.30
l-Ornithine	1.35
l-Serine	3.70
l-Threonine	4.60
l-Cysteine	0.59
l-Tyrosine	0.67
l-Histidine	4.70
l-Arginine	8.80
l-Aspartic acid	2.50
l-Asparagine	0.48
l-Glutamic acid	5.70
l-lysine acetate	10.60
Total amino acids	100

Osmotic pressure: approximately 875 mOsm/L

## Additional file

Additional file 1: Table S1. It is a five-point scale for shivering grade assessment, published by Wrench IJ et al in *Anaesthesia* on 1997. (DOC 31 kb)
