# Supplementary material for: Intraoperative infusion of branched-chain amino acids in patients undergoing gastrointestinal tumor surgery
Source: World J Surg Oncol. 2015 Dec 15;13:336. doi: 10.1186/s12957-015-0751-y (PMC4678463; doi:10.1186/s12957-015-0751-y)
Supplement: Additional file 1: Table S1. — It is a five-point scale for shivering grade assessment, published by Wrench IJ et al in Anaesthesia on 1997. (DOC 31 kb) [file 12957_2015_751_MOESM1_ESM.doc]

Additional file 1: Table S1．Wrench grading system for post-anesthetic shivering[^1^](#_ENREF_1)

| Grade | Clinical manifestation |
| --- | --- |
| 0 | no shivering |
| 1 | no visible muscle activity, but piloerection, peripheral vasoconstriction, or both are present (other causes excluded) |
| 2 | muscular activity in only one muscle group |
| 3 | moderate muscular activity in more than one muscle group, but no generalized shaking |
| 4 | violent muscular activity that involves the whole body |

1. Wrench IJ, Singh P, Dennis AR, Mahajan RP, Crossley AW: The minimum effective doses of pethidine and doxapram in the treatment of post-anaesthetic shivering. Anaesthesia 1997; 52: 32-6
